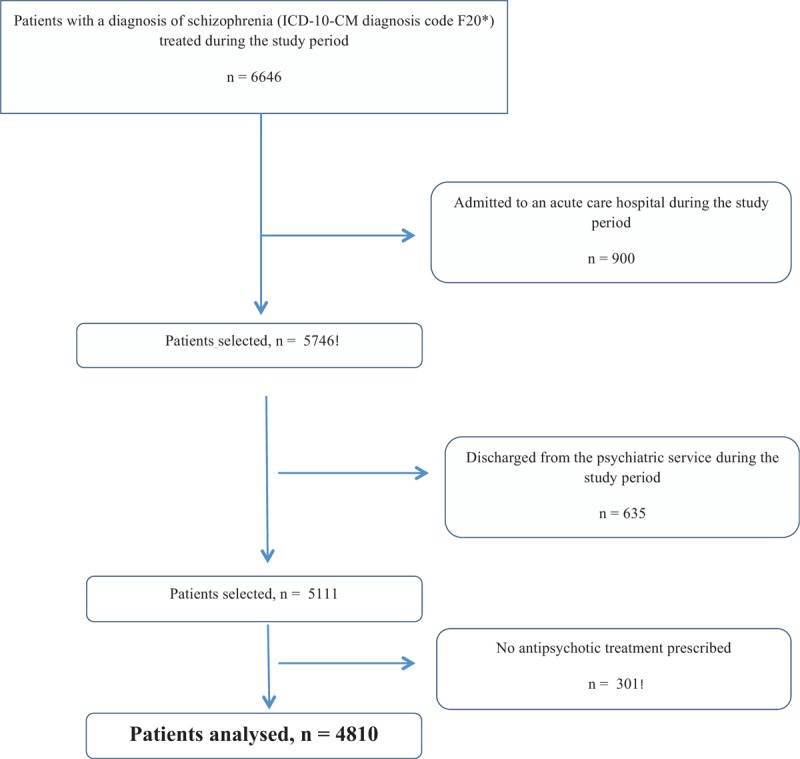# Influence of an inconsistent appearance of antipsychotics on drug adherence in patients with schizophrenia: Erratum

**DOI:** 10.1097/MD.0000000000013496

**Published:** 2018-11-21

In the article, “Influence of an inconsistent appearance of antipsychotics on drug adherence in patients with schizophrenia”,^[1]^ which appears in Volume 97, Issue 44 of *Medicine*, Figure 1 is in correct and should be:

**Figure:**